# Content analysis of PROMIS physical function banks using the international classification of functioning, disability and health

**DOI:** 10.1007/s11136-025-04071-1

**Published:** 2025-09-25

**Authors:** Michelle M. Langer, Kathryn C. Nesbit, Alex Dien, Theresa Jaramillo, Sang S. Pak

**Affiliations:** 1 Department of Medical Social Sciences, Northwestern University Feinberg School of Medicine, Chicago, IL USA; 2 Department of Physical Therapy, San Francisco State University, San Francisco, CA USA; 3 Department of Physical Therapy and Rehabilitation Services, University of California San Francisco, San Francisco, CA USA

**Keywords:** PROMIS physical function, International classification of functioning (ICF), Content validity, Patient-centered care, Functional measurement

## Abstract

**Purpose:**

This study links the current version of the PROMIS Physical Function bank, including its Mobility and Upper Extremity sub-banks, to the World Health Organization’s International Classification of Functioning, Disability and health (ICF). This research aims to enhance interpretability and applicability of these measures across healthcare and research settings by establishing a common language between PROMIS and ICF.

**Methods:**

165 items from PROMIS Physical Function v2.0, Mobility v2.1, and Upper Extremity v2.1 banks were mapped to ICF codes at primary, second, and third levels. Two investigators independently coded items, with a referee for consensus. Inter-rater reliability was assessed to evaluate coder agreement, and content composition was analyzed through frequencies of mapped ICF codes.

**Results:**

Mapping achieved substantial inter-rater reliability (α = 0.79–0.88). "D4 Mobility" was the most represented ICF chapter across banks, with the Mobility bank having the highest proportion (93%). The Physical Function bank covered seven primary ICF chapters, dominated by "D4 Mobility" (69%) and "D5 Self-care" (12%). The Mobility and Upper Extremity banks showed distinct content focuses within the broader physical function domain.

**Conclusion:**

This analysis reveals broad ICF concept coverage in PROMIS Physical Function banks, primarily of Mobility and Self-care. The mapping enables clinicians to understand PROMIS content using familiar ICF terminology and supports goal-setting that integrates both frameworks. These capabilities enable innovative clinical care and customized measurement approaches. While acknowledging limitations, this work provides a foundation for integrated, patient-centered care and outcomes research.

**Supplementary Information:**

The online version contains supplementary material available at 10.1007/s11136-025-04071-1.

## Introduction

The increasing complexity of health terminologies, biomedical ontologies, classification systems, and measurement networks necessitates common frameworks for understanding and communicating health-related information. In response, a growing number of health measures have been linked to the World Health Organization's (WHO) International Classification of Functioning, Disability and Health (ICF) [1–4]. The ICF provides a standardized, internationally comparable system for describing and classifying health, functioning, and disability. The ICF conceptualizes disability and functioning as outcomes of interactions between health conditions (e.g., diseases, injuries) and contextual factors (environmental, physical, social, attitudinal, and personal influences) [5]. This comprehensive framework comprises four main components of classification and four hierarchical sub-levels of increasingly specific function and disability codes, offering a nuanced approach to characterizing health states.

In parallel with the ICF, the Patient Reported Outcome Measurement Information System (PROMIS) has emerged as a significant tool in person-centered health assessment. PROMIS evaluates and monitors physical, mental, and social health in both adults and children [6]. With 3,250 items covering 161 self-reported health domains, PROMIS represents the world's largest patient-reported outcome (PRO) measurement system. While a 2014 study linked many PROMIS adult PRO measures to the ICF at the second level of classification specificity [7], the current version of the PROMIS Physical Function v2.0, one of the most widely used PROMIS measures comprising 165 items, was not available at that time. This new version of the bank includes 41 additional items, representing a substantial expansion that warranted comprehensive ICF mapping to support current clinical and research applications. This bank also includes two sub-banks that are also widely used: PROMIS Mobility v2.1 and PROMIS Upper Extremity v2.1.

Linking these PROMIS measures to the ICF offers several potential benefits. It establishes a common language for research and clinical care, enhancing communication across disciplines while introducing familiarity for clinicians in ICF-integrated settings such as physical therapy and orthopedics. For example, research has shown the value of ICF-based content analysis for instrument selection decisions, but detailed ICF mapping of current PROMIS measures is needed to support such applications in practice [8]. Additionally, this integration can facilitate more nuanced assessments of patient functioning that guide both procedural interventions and patient education by identifying deficits that clinicians may not routinely address due to time constraints in clinical encounters.

This manuscript aims to provide a comprehensive item-mapping and content analysis of the current versions of the PROMIS Physical Function, PROMIS Mobility, and PROMIS Upper Extremity banks. Items are classified to the third level of ICF specificity, and multiple ICF classifications are utilized when items measure multiple concepts, ensuring a thorough account of the banks' content. Additionally, the content composition of the Mobility and Upper Extremity sub-banks is explored and compared against the full PROMIS Physical Function bank. By bridging these two influential systems—ICF and PROMIS—this work seeks to enhance the interpretability, applicability, and integration of patient-reported outcomes in diverse healthcare and research settings.

## Methods

### Measures

The PROMIS Physical Function v2.0 bank (PROMIS PF) [9, 10] contains 165 items and measures self-reported physical function capability rather than actual performance of physical activities. Each item describes activities and/or physical movements in which respondents’ self-reported ability/capability is scored using a Likert scale. This includes the functioning of one’s upper extremities (dexterity), lower extremities (walking or mobility), and central regions (neck, back), as well as instrumental activities of daily living, such as running errands. The PROMIS Mobility v2.1 bank (PROMIS MOB) [11] and the PROMIS Upper Extremity v2.1 bank (PROMIS UE) [12] are each a subset of PROMIS PF. Each PROMIS PF instrument is appropriate for the adult general population as well as adults with chronic health conditions.

PROMIS MOB and PROMIS UE contain items that are mutually exclusive of one another. PROMIS MOB contains 44 items and focuses on activities of physical mobility, such as getting out of bed/chair and running. PROMIS UE contains 46 items and targets activities that require use of an upper extremity, including shoulder, arm, and hand activities; examples include writing, using buttons, and opening containers.

### Mapping of PROMIS items to ICF codes:

The (ICF) is a standardized framework developed by the WHO to describe and classify function, disability, and health [5]. Officially endorsed by WHO members in 2001, it led to the development of the ICF browser for clinicians, researchers, and administrators [13]. The ICF classification structure consists of components denoted by letters (b, s, d, e), followed by numeric codes for hierarchical levels. The primary level comprises chapters (one digit, 34 total), followed by second (two additional digits, 362 categories), third, and fourth levels (one additional digit each, 1424 + fourth-level categories). For example, the code “d450” refers to “walking” (a second-level category) within the “d4: Mobility” chapter of the Activities and Participation component. More detailed classifications, such as “d4500” (walking short distances), represent third-level specificity. The ICF is divided into two parts: Part 1 covers functioning and disability, including body functions (b), body structures (s), and activities and participation (d); Part 2 covers contextual factors, including environmental factors (e) and personal factors.

For this study, the term "concept" was used to refer to the underlying idea or construct represented by an item or ICF category, allowing for a conceptual mapping between PROMIS items and ICF categories.

We used the refined ICF linking rules as outlined by Cieza et al. [14] to map each PROMIS PF item to ICF code(s), following a similar approach to previous studies [1, 7, 15]. Two ICF content experts (SP, CN), each with over 20 years of clinical experience using the ICF framework, independently mapped the 165 items from the PF bank. Prior to mapping, the experts trained themselves with the online ICF browser and its detailed categorization. Using a prepopulated table of all PF items, experts mapped each item to the most appropriate ICF chapter(s). When applicable, they identified secondary ICF chapters that also fit the item content. The resulting mapped ICF codes were cross-compared (0 = not matched; 1 = matched). Through an iterative process involving four meetings, the investigators reviewed each item bank to reach consensus on:

1. Primary PROMIS concepts measured
2. Primary ICF chapter
3. Second and third-level ICF categories, when applicable

When experts assigned different ICF chapters (primary or secondary), these disagreements were recorded in a tracking spreadsheet for systematic resolution. For each disputed item, experts discussed their rationale and reached agreement by focusing on the item’s primary functional concept. In cases of persistent disagreement, a referee (TJ) intervened to establish consensus by reviewing the PROMIS items and ICF codes to derive a final decision.

Similar to Tucker et al.'s mapping approach, the primary concept in each PROMIS item was first identified and then the ICF browser was used to match it to the appropriate ICF chapter and more specific categories when possible. For complex items, the following strategies were used:

1. Multi-concept items: When an item contained multiple concepts, the primary concept and, if necessary, a secondary concept were coded. Differentiation was made between primary concepts and descriptive examples, often indicated by phrases like "such as" or "for example."
2. Shared second-level categories: When two concepts shared a second-level ICF category but differed at the third level, both the shared second-level and the distinct third-level categories were coded.
3. Use of "unspecified" codes: "Unspecified" third-level codes were used when an item implied a shared second-level concept but lacked a clear third-level match.
4. Concept identification: Primary concepts were identified by action verbs or nouns, often highlighted by examples in the item text.
5. Broad coding for varied examples: When items provided multiple examples of a concept, the highest applicable ICF level (typically third-level) that accurately encompassed all examples without losing the core concept was used.

This approach allowed for consistency in mapping while accommodating the complexity and variability of PROMIS items. It also ensured that the coding reflected both the specificity of individual items and their broader conceptual alignment with the ICF framework.

### Data analyses

Inter-rater reliability between the two ICF coders for each ICF level (chapter, second-level category, third-level category) was measured using Krippendorff's alpha (KA) for nominal values [16]. Gwet's probabilistic benchmarking method [17] was used to evaluate the extent of agreement between raters.

Frequencies of mapped ICF codes were computed at the chapter and second-level category levels for each PROMIS bank (Physical Function, Mobility, Upper Extremity).

All analyses were conducted using STATA Statistics V17 (StataCorp, USA), with complementary visualization performed using Tableau Software, LLC.

## Results

### Inter-rater reliability

The PROMIS PF, MOB, and UE banks were successfully mapped to ICF codes using an iterative consensus process by the two ICF coders with systematic resolution of disagreements and occasional referee input to achieve final consensus. Inter-rater reliability between the two ICF coders was sufficiently high with KAs ranging from 0.73 to 0.88. KA for the ICF chapter level was 0.79 (97.6% agreement), benchmarked as *Substantial*. KA for the second level ICF categories was 0.88 (89.1% agreement), benchmarked as *Almost Perfect*. KA for the third level ICF categories was 0.73 (73.9% agreement), benchmarked as *Substantial*.

### Primary level ICF distribution

**Fig. 1:**
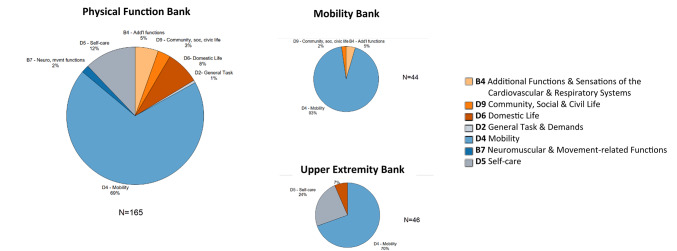
Primary international classification of functioning, health and disability (ICF) codes by PROMIS bank

**Fig. 2:**
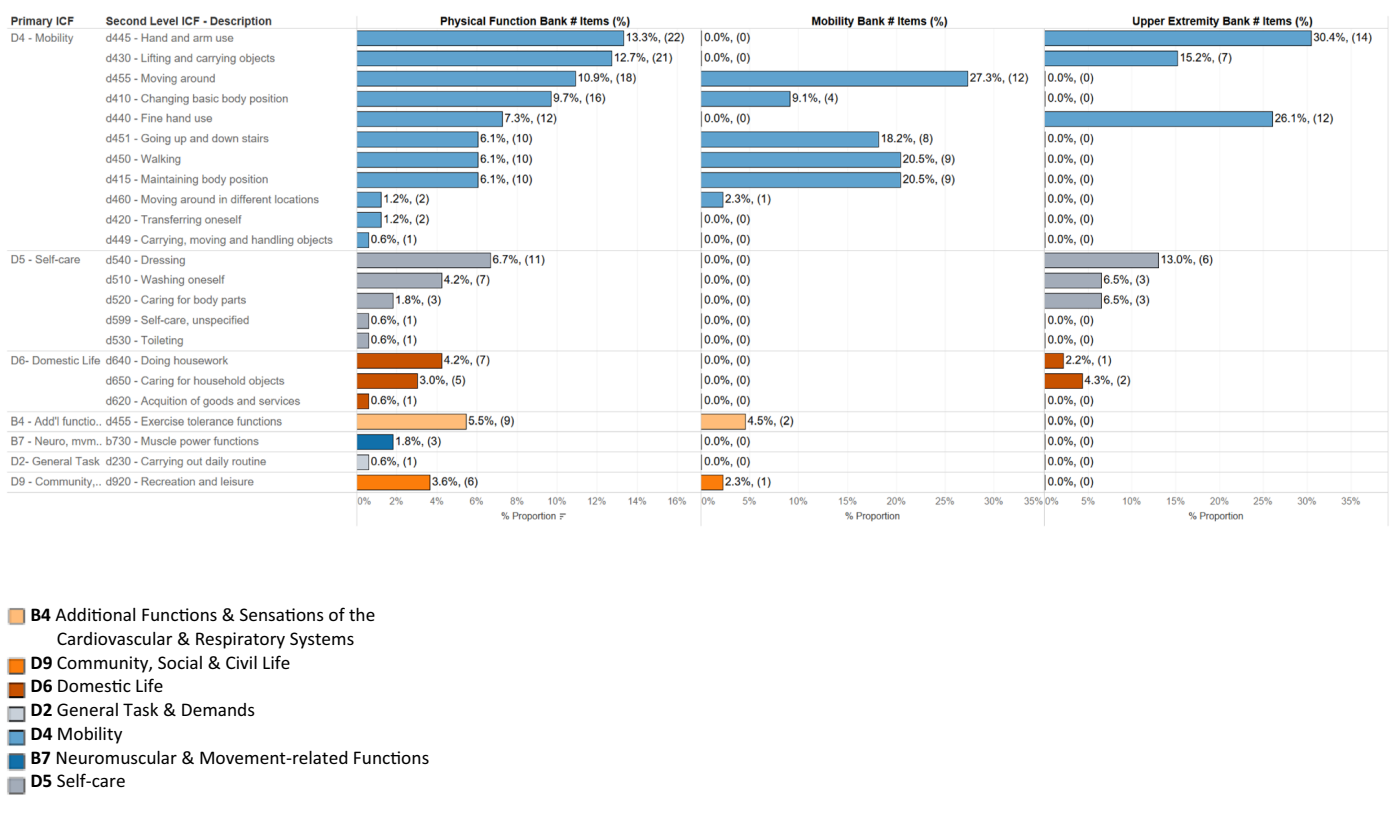
Second level international classification of functioning, health and disability (ICF) codes by PROMIS bank

Figure 1 displays the proportion of items for each PF bank characterized by the primary ICF chapter. The size of each pie chart is relative to the number of items in each bank. Across all banks, the "D4 Mobility" chapter represents the largest proportion of items. The MOB bank has the highest proportion of "D4 Mobility" items (93%) compared to PF and UE banks. The PF bank demonstrates the greatest diversity of ICF chapters, spanning from "D4 Mobility" (69% of items) to "D2 General Tasks and Demands" (1% of items). The "D5 Self-care" chapter represents the second-largest proportion of items for both the PF (12%) and MOB (24%) banks (Fig. 2).

### Second level ICF distribution

Table S1 further characterizes the items in each PF bank by the second level ICF categories. Within the "D4 Mobility" chapter, the PF bank encompasses a wide range of second level categories, including those represented in both the MOB and UE banks. The MOB bank contributes 43 items across six second level categories (“d455 Moving around”, “d410 Changing basic body position”, “d451 Going up and down stairs”, “d450 Walking”, “d415 Maintaining body position”, and “d460 Moving around in different locations”), while the UE bank contributes 33 items across three categories ("d445 Hand and arm use", "d430 Lifting and carrying objects", and “d440 fine hand use”). The PF bank also includes additional items in two second level categories (“d420 Transferring oneself” and “d449 Carrying, moving and handling objects”) not represented in the MOB or UE banks.

The "D5 Self-care" chapter has moderate representation in both the PF and UE banks, with 23 items in the PF bank (including 12 items from the UE bank) across three second level categories (“d450 Dressing”, “d510 Washing oneself”, and “d520 Caring for body parts”). The PF bank includes two additional second level categories (d599 Self-care, unspecified” and “d530 Toileting”) in this chapter not found in the UE bank.

The "D6 Domestic Life" chapter has minor representation in both the PF and UE banks, with 12 items in the PF bank (including 3 items from the UE bank) across two second level categories (“d460 Doing housework” and “d650 Caring for household objects”). The PF bank includes one additional second level category (“d620 Acquisition of goods and services”) in this chapter not found in the UE bank.

The PF and MOB banks also include a small number of items (9 items in the PF bank, including 2 items in the MOB bank) from the “B4 Additional Functions & Sensations of the Cardiovascular & Respiratory Systems” chapter with the second level category of “b455 Exercise tolerance functions”. These banks also include a small number of items (6 items in the PF bank, including 1 item in the MOB bank) coded within the “D9 Community, Social, & Civil Life” chapter with the second level category of “d920 Recreation and leisure”.

Unique to the PF bank (not included in the MOB or UE banks) are items coded under two additional ICF chapters: "B7 Neuromusculoskeletal and movement-related functions" (3 items) and "D2 General tasks and demands" (1 item). This includes three items with a second level category of “b730 Muscle power functions” and one item with a second level category of “d230 Carrying out daily routine”.

Full characterization of the items down to the third level category is included in the Supplementary Materials to provide further detail regarding the content coverage of these PROMIS banks.

## Discussion

This study presents the first comprehensive item-mapping and content analysis of current versions of PROMIS Physical Function, PROMIS Mobility, and PROMIS Upper Extremity banks using the ICF framework, extending Tucker et al.’s foundational PROMIS-ICF mapping [7]. The methodological approach, which included strategies for coding complex PROMIS items and an iterative coding process, resulted in high inter-rater reliability between coders. The content analysis revealed that the PROMIS banks cover a broad spectrum of ICF concept, with a primary focus on Mobility. Self-care emerged as the second most represented ICF chapter, followed by Domestic Life and Community, Social, and Civil Life. This comprehensive mapping is particularly valuable given that physical function limitations significantly impact daily activities, social participation, and quality of life, making precise content understanding essential for effective clinical implementation of these measures [18, 19]. This detailed characterization provides clinicians with valuable guidance in selecting measures that best align with their specific needs and patient populations.

The mapping of these widely used PROMIS measures to ICF codes establishes a common language for researchers and clinicians across various disciplines [20–23]. This is particularly significant in rehabilitation science, where the ICF model has been officially endorsed by organizations such as the American Physical Therapy Association [24]. Physical therapists and other healthcare professionals who have integrated the ICF framework into their clinical practice [25–27] can now more readily understand and utilize PROMIS measures. This alignment with familiar ICF terminologies enhances the validity and accessibility of PROMIS measures, especially for clinicians and interdisciplinary team members who may be new to these tools.

Furthermore, this ICF-PROMIS alignment facilitates broader adoption of PROMIS measures, offering numerous advantages for population health management, value-based care initiatives, and patient-centered approaches [28–30]. The common conceptual framework provided by this mapping can enhance communication among healthcare providers, researchers, and policymakers, potentially leading to more cohesive and effective healthcare strategies.

The linkage between the ICF model and PROMIS measures also opens up innovative possibilities for clinical care. For instance, custom PROMIS measures can be created by selecting items that assess specific ICF concepts, allowing for tailored measurement while maintaining comparability across different impairments and chronic conditions. This customization potential could be particularly valuable in specialized clinical settings or for specific patient populations.

Moreover, clinical activities that typically employ ICF codes can now seamlessly incorporate PROMIS measures, streamlining care processes and potentially reducing patient burden. As an example, goal-setting practices can integrate ICF codes to identify target functions alongside PROMIS scores to specify desired functional levels. PROMIS T-score maps are particularly well-suited for this type of integrated approach [31], offering a quantitative dimension to the qualitative ICF framework.

While this study provides valuable insights, it is important to acknowledge its limitations and identify directions for future research. The coding process focused on key concepts within items, potentially omitting nuanced content that did not align directly with ICF codes. Our scope was also limited to PROMIS Physical Function banks, while other PROMIS domains remain to be linked to the ICF. Moreover, this study does not explicitly address the application of this mapping to clinical decision-making, an area for future research. Subsequent studies should investigate the practical implementation of this ICF-PROMIS alignment across diverse clinical settings and evaluate its impact on patient outcomes. Furthermore, exploring how ICF mapping might inform the development of future PROMIS measures represents a promising avenue for inquiry, potentially enhancing the measures' utility and relevance.

In conclusion, this study's ICF-based characterization of key PROMIS measures offers a foundation for enhanced understanding and utilization of these tools across various healthcare settings. By bridging the gap between the ICF framework and PROMIS measures, a path is laid for more integrated, patient-centered care and outcomes research. As healthcare continues to evolve towards more person-centered and value-based models, the synergy between ICF and PROMIS could play a crucial role in advancing both clinical practice and health services research.

## Supplementary Information

Below is the link to the electronic supplementary material.

Supplementary file1 (DOCX 49 kb)
