# Supplementary material for: Content analysis of PROMIS physical function banks using the international classification of functioning, disability and health
Source: Qual Life Res. 2025 Sep 25;34(11):3159–65. doi: 10.1007/s11136-025-04071-1 (PMC12681486; doi:10.1007/s11136-025-04071-1)
Supplement: Supplementary file 1 — Supplementary file1 (DOCX 49 kb) [file 11136_2025_4071_MOESM1_ESM.docx]

**Table 1. Supplementary Materials**

| **Item ID^[[1]](#footnote-1)^** | **Mobility Bank** | **Upper Extremity Bank** | **Item Concepts** | **ICF Component** | **Primary ICF** | **Second Level ICF** | **Second Level ICF – 2** | **Second Level ICF – 3** | **Third Level ICF** | **Third Level ICF - 2** |
| --- | --- | --- | --- | --- | --- | --- | --- | --- | --- | --- |
| Global06 | 0 | 0 | everyday physical activities: walking, climbing stairs, carrying groceries, moving chair | Activities and Participation (D) | d4 | d450 | d455 |  |  | d4551 |
| PFA1 | 0 | 0 | health limiting in vigorous activities: running, lifting heavy objects, strenuous sports | Activities and Participation (D) | d4 | d455 | d430 | d920 | d4552 | d4300 |
| PFA10 | 1 | 0 | Stand for one hour | Activities and Participation (D) | d4 | d415 |  |  | d4154 |  |
| PFA11 | 0 | 0 | Chores: vacuuming, yardwork | Activities and Participation (D) | d6 | d640 |  |  | d6409 |  |
| PFA12 | 0 | 0 | Pushing to open heavy door | Activities and Participation (D) | d4 | d445 |  |  | d4451 |  |
| PFA13 | 0 | 0 | Exercise for one hour | Body Functions (B) | b4 | b455 |  |  | b4558 |  |
| PFA14r1 | 0 | 1 | Carry heavy objects | Activities and Participation (D) | d4 | d430 |  |  | d4308 |  |
| PFA15 | 1 | 0 | Stand up from armless chair | Activities and Participation (D) | d4 | d410 |  |  | d4104 |  |
| PFA16r1 | 0 | 1 | Dressing, tying shoelaces, buttoning clothes | Activities and Participation (D) | d5 | d540 |  |  | d5408 |  |
| PFA17 | 0 | 1 | Reaching high cupboard | Activities and Participation (D) | d4 | d445 |  |  | d4452 |  |
| PFA18 | 0 | 1 | Hammer to pound nail | Activities and Participation (D) | d6 | d650 |  |  | d6508 |  |
| PFA19r1 | 1 | 0 | Run or job for two miles | Body Functions (B) | b4 | b455 |  |  | b4558 |  |
| PFA2 | 0 | 0 | Health limiting in exercising regularly | Body Functions (B) | b4 | b455 |  |  |  |  |
| PFA20 | 0 | 1 | Cutting food with utensils | Activities and Participation (D) | d4 | d445 |  |  | d4458 |  |
| PFA21 | 1 | 0 | Walk up and down stairs at normal pace | Activities and Participation (D) | d4 | d451 |  |  |  |  |
| PFA23 | 1 | 0 | Walk at least 15 mins | Body Functions (B) | b4 | b455 |  |  | b4558 |  |
| PFA27 | 1 | 0 | Run on uneven ground | Activities and Participation (D) | d4 | d455 |  |  | d4552 |  |
| PFA28 | 0 | 1 | Using hand to open a can with can opener | Activities and Participation (D) | d4 | d440 |  |  | d4408 |  |
| PFA29r1 | 0 | 1 | Pull heavy objects | Activities and Participation (D) | d4 | d445 |  |  | d4450 |  |
| PFA3 | 1 | 0 | Health limiting in bending, kneeling, or stooping | Activities and Participation (D) | d4 | d410 | d410 |  | d4105 | d4102 |
| PFA30 | 1 | 0 | Step up and down on curbs | Activities and Participation (D) | d4 | d451 |  |  |  |  |
| PFA31r1 | 0 | 0 | Independently getting up from floor from lying on back | Activities and Participation (D) | d4 | d410 |  |  | d4100 |  |
| PFA32 | 1 | 0 | Stand with knees straight | Activities and Participation (D) | d4 | d415 |  |  | d4154 |  |
| PFA33 | 0 | 0 | Exercise for half an hour | Body Functions (B) | b4 | b455 |  |  | b4558 |  |
| PFA34 | 0 | 1 | Wash your back | Activities and Participation (D) | d5 | d510 |  |  | d5100 |  |
| PFA35 | 0 | 1 | Open and close zipper | Activities and Participation (D) | d4 | d440 |  |  | d4408 |  |
| PFA36 | 0 | 1 | Putting on and taking off coat or jacket | Activities and Participation (D) | d5 | d540 | d540 |  | d5400 | d5401 |
| PFA37 | 1 | 0 | Stand for short period | Activities and Participation (D) | d4 | d415 |  |  | d4154 |  |
| PFA38 | 0 | 1 | Dry back with towel | Activities and Participation (D) | d5 | d510 |  |  | d5102 |  |
| PFA39r1 | 1 | 0 | run at fast pace for two miles | Activities and Participation (D) | d4 | d455 |  |  | d4552 |  |
| PFA4 | 0 | 0 | Health limiting around the house: scrubbing floors, lifting, moving heavy furniture | Activities and Participation (D) | d6 | d640 |  |  | d6408 |  |
| PFA40 | 0 | 1 | Turn key in lock | Activities and Participation (D) | d4 | d440 |  |  | d4408 |  |
| PFA41 | 1 | 0 | Squat and get up | Activities and Participation (D) | d4 | d410 |  |  | d4101 |  |
| PFA42 | 0 | 0 | Carry laundry basket a flight of stairs | Activities and Participation (D) | d4 | d430 |  |  | d4308 |  |
| PFA43r1 | 0 | 1 | Write with pen or pencil | Activities and Participation (D) | d4 | d440 |  |  | d4402 |  |
| PFA44 | 0 | 1 | Put on shirt or blouse | Activities and Participation (D) | d5 | d540 |  |  | d5400 |  |
| PFA45 | 0 | 0 | Get out of bed into chair | Activities and Participation (D) | d4 | d420 |  |  | d4200 |  |
| PFA47 | 0 | 0 | Pull on trousers | Activities and Participation (D) | d5 | d540 |  |  | d5400 |  |
| PFA48 | 0 | 1 | Peel fruit | Activities and Participation (D) | d4 | d440 |  |  | d4408 |  |
| PFA49 | 0 | 0 | Bend or twist back | Activities and Participation (D) | d4 | d410 | d410 |  | d4105 | d4108 |
| PFA5 | 0 | 0 | Health limiting lifting or carrying groceries | Activities and Participation (D) | d4 | d430 | d430 |  | d4300 | d4308 |
| PFA50 | 0 | 1 | Brush your teeth | Activities and Participation (D) | d5 | d520 |  |  | d5201 |  |
| PFA51 | 0 | 0 | Sit on edge of bed | Activities and Participation (D) | d4 | d415 |  |  | d4153 |  |
| PFA52 | 0 | 0 | Tie your shoelaces | Activities and Participation (D) | d5 | d540 |  |  | d5408 |  |
| PFA53 | 0 | 0 | Run errands and shop | Activities and Participation (D) | d6 | d620 |  |  | d6200 |  |
| PFA54 | 0 | 1 | Button shirt | Activities and Participation (D) | d5 | d540 |  |  | d5400 |  |
| PFA55 | 0 | 0 | Wash and dry body | Activities and Participation (D) | d5 | d510 |  |  | d5108 |  |
| PFA56 | 0 | 0 | Get in and out of car | Activities and Participation (D) | d4 | d410 |  |  | d4108 |  |
| PFA6 | 0 | 0 | Health limiting  bathing or dressing self | Activities and Participation (D) | d5 | d510 | d540 |  | d5101 | d5409 |
| PFA8 | 0 | 0 | Move chair from one root to another | Activities and Participation (D) | d4 | d430 |  |  |  |  |
| PFA9 | 0 | 0 | Bend down and pick up clothing from floor | Activities and Participation (D) | d4 | d410 | d449 |  | d4105 |  |
| PFB1 | 0 | 0 | Health limit in activities: vacuuming, sweeping floors, carrying in groceries | Activities and Participation (D) | d6 | d640 |  |  | d6409 |  |
| PFB10 | 1 | 0 | Climb up five steps | Activities and Participation (D) | d4 | d451 |  |  |  |  |
| PFB11 | 0 | 1 | Wash dishes, pots, utensils by using hand while standing | Activities and Participation (D) | d6 | d640 |  |  | d6401 |  |
| PFB12 | 0 | 0 | Make bed, spread and tuck bed sheets | Activities and Participation (D) | d6 | d640 |  |  | d6408 |  |
| PFB13 | 0 | 1 | Carry shopping bag or briefcase | Activities and Participation (D) | d4 | d430 |  |  | d4308 |  |
| PFB14 | 0 | 0 | Take bath in tub | Activities and Participation (D) | d5 | d510 |  |  | d5101 |  |
| PFB15r1 | 0 | 1 | Change light bulb | Activities and Participation (D) | d4 | d445 |  |  | d4453 |  |
| PFB16r1 | 0 | 1 | Using index finger to press an object | Activities and Participation (D) | d4 | d440 |  |  | d4409 |  |
| PFB17 | 0 | 0 | Put on and off socks | Activities and Participation (D) | d5 | d540 | d540 |  | d5402 | d5403 |
| PFB18 | 0 | 1 | Shave facial hair or put makeup | Activities and Participation (D) | d5 | d520 |  |  | d5200 |  |
| PFB19r1 | 0 | 1 | Squeeze tube of toothpaste | Activities and Participation (D) | d4 | d440 |  |  | d4408 |  |
| PFB20r1 | 0 | 1 | Cut piece of paper with scissors | Activities and Participation (D) | d4 | d440 |  |  | d4402 |  |
| PFB21r1 | 0 | 1 | Pickup coins from table | Activities and Participation (D) | d4 | d440 |  |  | d4400 |  |
| PFB22 | 0 | 1 | Hold plate full of food | Activities and Participation (D) | d4 | d430 |  |  | d4308 |  |
| PFB23r1 | 0 | 1 | Pour liquid from bottle into glass | Activities and Participation (D) | d4 | d445 |  |  | d4458 |  |
| PFB24 | 1 | 0 | Run short distance | Activities and Participation (D) | d4 | d455 |  |  | d4552 |  |
| PFB25 | 0 | 1 | Push to open door after turning the knob | Activities and Participation (D) | d4 | d445 | d445 |  | d4451 | d4453 |
| PFB26 | 0 | 1 | Shampoo hair | Activities and Participation (D) | d5 | d510 |  |  | d5100 |  |
| PFB27 | 0 | 1 | Tie knot or bow | Activities and Participation (D) | d4 | d440 |  |  | d4402 |  |
| PFB28r1 | 0 | 1 | Lift 10 pounds above shoulder | Activities and Participation (D) | d4 | d430 |  |  | d4300 |  |
| PFB29r1 | 0 | 0 | Lift full cup or glass to mouth | Activities and Participation (D) | d4 | d430 |  |  | d4300 |  |
| PFB3 | 0 | 0 | Putting trash bag outside | Activities and Participation (D) | d6 | d640 |  |  | d6405 |  |
| PFB30 | 0 | 1 | Open milk carton | Activities and Participation (D) | d4 | d440 |  |  | d4408 |  |
| PFB31r1 | 0 | 1 | Open car door | Activities and Participation (D) | d4 | d445 |  |  | d4458 |  |
| PFB32 | 1 | 0 | Stand unsupported for 10 mins | Activities and Participation (D) | d4 | d415 |  |  | d4154 |  |
| PFB33 | 0 | 1 | Reach back pocket to remove item | Activities and Participation (D) | d4 | d445 |  |  | d4452 |  |
| PFB34 | 0 | 1 | Change light bulb overhead | Activities and Participation (D) | d4 | d445 |  |  | d4458 |  |
| PFB36 | 0 | 1 | Put on pullover sweater | Activities and Participation (D) | d5 | d540 |  |  | d5400 |  |
| PFB37r1 | 0 | 1 | Turn faucets on and off | Activities and Participation (D) | d4 | d445 |  |  | d4453 |  |
| PFB39r1 | 0 | 1 | Reach and bring down 5 pounds object above head | Activities and Participation (D) | d4 | d445 |  |  | d4452 |  |
| PFB4 | 0 | 0 | Dancing for half an hour | Activities and Participation (D) | d9 | d920 |  |  | d9202 |  |
| PFB40 | 1 | 0 | Stand up on tiptoes | Activities and Participation (D) | d4 | d415 |  |  | d4158 |  |
| PFB41 | 0 | 1 | Trim fingernails | Activities and Participation (D) | d5 | d520 |  |  | d5203 |  |
| PFB42 | 1 | 0 | Stand unsupported for 30 mins | Activities and Participation (D) | d4 | d415 |  |  | d4154 |  |
| PFB43 | 0 | 0 | Health limiting self care activities: dressing, combing hair, toileting, eating bathing | Activities and Participation (D) | d5 | d599 |  |  |  |  |
| PFB44 | 0 | 0 | Health limiting activities: moving table, push vacuum cleaner, playing goft | Body Functions (B) | b4 | b455 |  |  |  |  |
| PFB45 | 0 | 0 | Health limiting in sports like swimming or bowling | Activities and Participation (D) | d9 | d920 |  |  | d9201 |  |
| PFB48 | 0 | 0 | Health limiting taking shower | Activities and Participation (D) | d5 | d510 |  |  | d5109 |  |
| PFB49 | 1 | 0 | Health limiting short walk less than 15 mins | Activities and Participation (D) | d4 | d450 |  |  | d4500 |  |
| PFB50 | 0 | 0 | Health limiting with daily physical activities | Activities and Participation (D) | d2 | d230 |  |  | d2302 |  |
| PFB51 | 0 | 0 | Health limiting sports like swimming, tennis, basketball | Activities and Participation (D) | d9 | d920 |  |  | d9201 |  |
| PFB54 | 0 | 0 | Health limiting from going outside home | Activities and Participation (D) | d4 | d460 |  |  | d4602 |  |
| PFB56r1 | 0 | 1 | Lift one pound to shoulder level without bending elbow | Activities and Participation (D) | d4 | d430 |  |  | d4308 |  |
| PFB5r1 | 1 | 0 | Health limiting hiking 2 miles on uneven surface, including hills | Activities and Participation (D) | d4 | d450 | d450 |  | d4502 | d4501 |
| PFB7 | 0 | 0 | Health limiting activating: backpacking, skiing, tennis, bicycling, jogging | Activities and Participation (D) | d9 | d920 |  |  | d9209 |  |
| PFB8r1 | 0 | 0 | Carry two full groceries bags for 100 yards | Activities and Participation (D) | d4 | d430 |  |  | d4308 |  |
| PFB9 | 1 | 0 | Jump up and down | Activities and Participation (D) | d4 | d455 |  |  | d4553 |  |
| PFC10 | 1 | 0 | Health limiting climbing flights of stairs | Activities and Participation (D) | d4 | d451 |  |  |  |  |
| PFC11 | 0 | 0 | Health limiting yard work: raking leaves, weeding, pushing lawn mower | Activities and Participation (D) | d6 | d640 |  |  | d6409 |  |
| PFC12 | 0 | 0 | Health limiting two hours of physical labor | Body Functions (B) | b4 | b455 |  |  |  |  |
| PFC13r1 | 1 | 0 | Run 100 yards | Activities and Participation (D) | d4 | d455 |  |  | d4552 |  |
| PFC21 | 1 | 0 | Run on even ground | Activities and Participation (D) | d4 | d455 |  |  | d4552 |  |
| PFC29 | 1 | 0 | Walk up and down two steps | Activities and Participation (D) | d4 | d451 |  |  |  |  |
| PFC30 | 0 | 0 | Carry suitcase to flight of stairs | Activities and Participation (D) | d4 | d430 | d451 |  | d4301 |  |
| PFC31 | 0 | 0 | Reach into low cupboard | Activities and Participation (D) | d4 | d445 |  |  | d4452 |  |
| PFC32 | 1 | 0 | Climb up 5 flights of stairs | Activities and Participation (D) | d4 | d451 |  |  |  |  |
| PFC33r1 | 1 | 0 | Run ten miles | Activities and Participation (D) | d4 | d455 |  |  | d4552 |  |
| PFC35 | 0 | 0 | Health limiting eight hours of physical labor | Body Functions (B) | b4 | b455 |  |  |  |  |
| PFC36r1 | 1 | 0 | Health limiting walking more than mile | Activities and Participation (D) | d4 | d450 |  |  | d4501 |  |
| PFC37 | 1 | 0 | Health limiting climbing one flight of stairs | Activities and Participation (D) | d4 | d451 |  |  |  |  |
| PFC38 | 1 | 0 | Walk normal pace | Activities and Participation (D) | d4 | d450 |  |  | d4508 |  |
| PFC39 | 1 | 0 | Stand without losing balance for several minutes | Activities and Participation (D) | d4 | d415 |  |  | d4154 |  |
| PFC40 | 1 | 0 | Kneel on floor | Activities and Participation (D) | d4 | d415 |  |  | d4152 |  |
| PFC41 | 0 | 0 | Get up and down from low soft couch | Activities and Participation (D) | d4 | d410 |  |  | d4103 |  |
| PFC42 | 0 | 0 | Open tight or new jar | Activities and Participation (D) | d4 | d445 |  |  | d4453 |  |
| PFC43 | 0 | 1 | Using hands to turn faucets, kitchen gadgets, sewing | Activities and Participation (D) | d4 | d440 |  |  | d4409 |  |
| PFC45r1 | 0 | 0 | Get up and down from toilet | Activities and Participation (D) | d4 | d410 |  |  | d4103 |  |
| PFC46 | 0 | 0 | Transfer back and forth from bed to chair | Activities and Participation (D) | d4 | d420 |  |  | d4200 |  |
| PFC47 | 0 | 0 | Get out of bed | Body Functions (B) | b4 | b455 |  |  | b4558 |  |
| PFC48 | 0 | 0 | Carry heavy household items up a fight of stairs | Activities and Participation (D) | d4 | d430 | d451 |  | d4309 |  |
| PFC49 | 0 | 1 | Water plants | Activities and Participation (D) | d6 | d650 |  |  | d6505 |  |
| PFC51 | 0 | 0 | Wipe yourself after using toilet | Activities and Participation (D) | d5 | d530 |  |  | d5308 |  |
| PFC52 | 0 | 0 | Turn from side to side in bend | Activities and Participation (D) | d4 | d410 |  |  | d4107 |  |
| PFC53 | 0 | 0 | Get in and out of bed | Activities and Participation (D) | d4 | d410 |  |  | d4100 |  |
| PFC54 | 0 | 0 | Health limiting get in and out of bathtub | Activities and Participation (D) | d4 | d410 |  |  | d4108 |  |
| PFC56 | 1 | 0 | Health limiting walking in house | Activities and Participation (D) | d4 | d460 |  |  | d4600 |  |
| PFC6r1 | 1 | 0 | Walk a block on flat ground | Activities and Participation (D) | d4 | d450 |  |  | d4500 |  |
| PFC7r1 | 1 | 0 | Run five miles | Activities and Participation (D) | d4 | d455 |  |  | d4552 |  |
| PFC8 | 0 | 1 | Health limiting opening previous opened jar | Activities and Participation (D) | d4 | d445 |  |  | d4453 |  |
| PFM1 | 0 | 0 | Dig 2-foot hole in dirt using shovel | Activities and Participation (D) | d4 | d445 |  |  | d4458 |  |
| PFM10 | 0 | 0 | Do a pull-up | Body Functions (B) | b7 | b730 |  |  | b7308 |  |
| PFM12 | 0 | 0 | Lift heavy object (20 lbs) above head | Activities and Participation (D) | d4 | d430 |  |  | d4308 |  |
| PFM15 | 0 | 0 | Hit backboard with basketball from free-throw line | Activities and Participation (D) | d4 | d445 |  |  | d4454 |  |
| PFM16 | 0 | 1 | Pass 20-pound food to others at the table | Activities and Participation (D) | d4 | d430 |  |  | d4308 |  |
| PFM17 | 0 | 0 | Remove heavy suitcase (50 lbs) from overhead bin on airplane or bus | Activities and Participation (D) | d4 | d430 |  |  | d4308 |  |
| PFM18 | 0 | 1 | Swing handheld sports items for five minutes | Activities and Participation (D) | d4 | d445 |  |  | d4458 |  |
| PFM19 | 0 | 0 | Consecutively do 10 sit-ups | Body Functions (B) | b7 | b730 |  |  | b7305 |  |
| PFM2 | 0 | 1 | Lift heavy painting or picture to hang on wall above eye-level | Activities and Participation (D) | d4 | d430 |  |  | d4308 |  |
| PFM21 | 1 | 0 | Consecutively climb stairs of 10 story building | Activities and Participation (D) | d4 | d451 |  |  |  |  |
| PFM23 | 1 | 0 | Brisk walk for 20 minutes straight | Activities and Participation (D) | d4 | d450 |  |  | d4508 |  |
| PFM25 | 1 | 0 | Come to complete stop after running | Activities and Participation (D) | d4 | d455 |  |  | d4558 |  |
| PFM26 | 1 | 0 | Make sharp turn while running fast | Activities and Participation (D) | d4 | d455 |  |  | d4558 |  |
| PFM27 | 0 | 0 | Consecutively jump rope for 10 minutes | Activities and Participation (D) | d4 | d455 |  |  | d4558 |  |
| PFM28 | 1 | 0 | Jump over 1 foot object | Activities and Participation (D) | d4 | d455 |  |  | d4558 |  |
| PFM29 | 0 | 0 | Jump over 3 feet wide puddle | Activities and Participation (D) | d4 | d455 |  |  | d4558 |  |
| PFM3 | 0 | 0 | Paint wall with brush or roller for 2 hours consecutively | Activities and Participation (D) | d6 | d650 |  |  | d6508 |  |
| PFM32 | 1 | 0 | Jump 2 feet | Activities and Participation (D) | d4 | d455 |  |  | d4558 |  |
| PFM33 | 1 | 0 | Walk across balance beam | Activities and Participation (D) | d4 | d450 |  |  | d4508 |  |
| PFM34 | 1 | 0 | Stand on one foot with eyes closed for 30 seconds | Activities and Participation (D) | d4 | d415 |  |  | d4158 |  |
| PFM35 | 1 | 0 | Walk in straight line in tandem for 5 yards | Activities and Participation (D) | d4 | d450 |  |  | d4508 |  |
| PFM36 | 0 | 0 | Put hands flat on floor with both feet flat on ground | Activities and Participation (D) | d4 | d410 |  |  | d4108 |  |
| PFM37 | 0 | 0 | Carry large baby (15 lbs) out of house to car | Activities and Participation (D) | d4 | d430 |  |  | d4308 |  |
| PFM38 | 0 | 0 | Lift and load one 50-pound bag of sand into car | Activities and Participation (D) | d4 | d430 |  |  | d4308 |  |
| PFM4 | 0 | 0 | Row boat for 30 mins consecutively | Activities and Participation (D) | d4 | d445 |  |  | d4458 |  |
| PFM40 | 0 | 0 | Climb 6-foot ladder | Activities and Participation (D) | d4 | d455 |  |  | d4551 |  |
| PFM43 | 0 | 0 | Push empty refrigerator forward 1 yard | Activities and Participation (D) | d4 | d445 |  |  | d4451 |  |
| PFM44 | 0 | 0 | Carry 50 lbs bag of sand for 25 yards | Activities and Participation (D) | d4 | d430 |  |  | d4308 |  |
| PFM46 | 0 | 0 | Pull sled or wagon with children (100 lbs) for 100 yards | Activities and Participation (D) | d4 | d445 |  |  | d4450 |  |
| PFM49 | 0 | 0 | Stand up from push-up positions five times quickly | Activities and Participation (D) | d4 | d410 |  |  | d4104 |  |
| PFM51 | 0 | 0 | Swim laps for 30 mins | Activities and Participation (D) | d4 | d455 |  |  | d4554 |  |
| PFM53 | 1 | 0 | Dance rigorously for one hour | Activities and Participation (D) | d9 | d920 |  |  |  |  |
| PFM6 | 0 | 0 | Hand wash and wax car for 2 hours without rest | Activities and Participation (D) | d6 | d650 |  |  | d6503 |  |
| PFM7 | 0 | 0 | 5 push-ups consecutively | Body Functions (B) | b7 | b730 |  |  | b7308 |  |
| PFM9 | 0 | 0 | Rake leaves or sweep for 1 hr without rest | Activities and Participation (D) | d6 | d650 |  |  | d6508 |  |

1. Complete item content for the PROMIS Physical Function v2.0, Mobility v2.1, and Upper Extremity v2.1 banks is available at HealthMeasures.net (https://www.healthmeasures.net). Copyright restrictions prevent reproduction of full item text in this manuscript. [↑](#footnote-ref-1)
